# Spatial Distribution of Nitrogen on Grazed Karst Landscapes

**DOI:** 10.1100/tsw.2001.374

**Published:** 2001-11-29

**Authors:** Douglas G. Boyer, Ghiath A. Alloush

**Affiliations:** Appalachian Farming Systems Research Center, USDA-ARS, Beaver, WV 25813, USA

**Keywords:** nitrate, ammonium, karst, sinkhole, grazing, manure

## Abstract

The impact on water quality by agricultural activity in karst terrain is an important consideration for resource management within the Appalachian region. Karst areas comprise about 18% of the region’s land area. An estimated one-third of the region’s farms, cattle, and agricultural market value are located on karst terrain. Mean nitrate concentrations in several karst springs in southeastern West Virginia exhibit a strong linear relationship with the percentage of agriculture land cover. Development of best management practices for efficient nitrogen (N) use and reduction of outflow of N to water from karst areas requires knowledge about N dynamics on those landscapes. Water extractable NO_3_-N and NH_4_-N were measured along transects at four soil depths in two grazed sinkholes and one wooded sinkhole. Distribution of soil NO_3_-N and NH_4_-N were related to frequency of animal presence and to topographic and hydrologic redistribution of soil and fecal matter in the grazed sinkholes. Karst pastures are characterized by under drainage and funneling of water and contaminants to the shallow aquifer. Control of NO_3_-N leaching from karst pasture may depend on management strategies that change livestock grazing behavior in sinkholes and reduce the opportunity for water and contaminants to quickly reach sinkhole drains.

## INTRODUCTION

Pasture management often results in an increase in organic matter content of the surface soil. The rate of organic matter accumulation and time taken to reach equilibrium, where organic matter additions are balanced by mineralization and losses, varies considerably with initial organic matter level, soil type, climate, and management[1]. Nitrogen (N) compounds (i.e., $NO_3$ and $NH_4$) do not necessarily accumulate at the same rate at a particular site, nor do they necessarily reach equilibrium at the same time[1,2]. Increasing N concentrations in surface soil layers of managed pastures is an environmental concern in humid southeastern West Virginia, where over 80% of the agricultural lands are in permanent pasture and limestone geology creates an intricate mix of surface and groundwater.

Continuous grazing and deposition of manure by grazing cattle in addition to fertilization with commercial fertilizer can raise the potential for pollution of surface and groundwater from pastures. Robbins[3], in a review article, concluded that pollution resulting from grazing cattle was related to hydrogeological and management factors and not to the number of animals or the amount of waste involved. Greater $NO_3$-N concentrations in surface runoff from fertilized grazed pastures have been reported when grazed pastures were compared to adjacent control areas[4,5,6,7,8].

The impact on water quality by pasture-based agricultural systems in karst landscapes is an important consideration for resource management in the Appalachian region. Karst areas constitute about 18% of the region's land area[9]. An estimated one-third of the region's farms, cattle, and agricultural enterprises are located on karst landscape[10]. Because of the interrupted surface drainage and conduit flow in mature karst terrain, a relatively rapid and sometimes direct connection exists between surface and groundwater[11]. Therefore, sources of contamination resulting from fertilization practices in karst landscape have led to rapid movement of $NO_3$-N into groundwater[12]. Elevated levels of $NO_3$-N have been reported in karst groundwater in West Virginia[13,14]. Recent study has attributed elevated levels of $NO_3$-N in karst groundwater in West Virginia to animal agriculture[15].

Information about $NH_4$-N distribution with soil depth on hill landscapes is inadequate. Ammonium-N usually results from mineralization of the organic matter in the soil and with consequent nitrification produces nitrate[2]. The ratio of $NH_4$-N to $NO_3$-N is often greater in grazed pasture and grasslands compared to arable soils[15,16,17,18]. Since the $NH_4^+$ molecule is a cation, it moves much slower with percolating water as compared to nitrate and, therefore, $NH_4$-N accumulates to higher concentrations relative to $NO_3$-N in the surface soil layers[16]. Thus, $NH_4$-N may constitute a significant portion of mineral N in surface runoff from managed pastures.

This investigation was carried out on typical karst pasture sinkhole topography in Greenbrier County, West Virginia. The objective of this study was to investigate the effects of grazing management on $NO_3$-N and $NH_4$-N distribution in surface soil layers across sinkholes.

## EXPERIMENTAL PROCEDURES

The study area, which is located in Greenbrier County in southern West Virginia (Fig. 1), is typical of pastured karst topography in central Appalachia. Soil on the floor of the sinkholes (Lindside silt loam) is a fine-loamy, mixed, active, mesic, Typic Eutrudepts and the soil on the sideslopes (Frankstown silt loam) is a fine-loamy, mixed, active, mesic, Typic Hapludalfs. Typical of karst soils, the soil depths were highly variable, ranging from 0 to >1 m in very short distances (i.e., <1 m). The study included

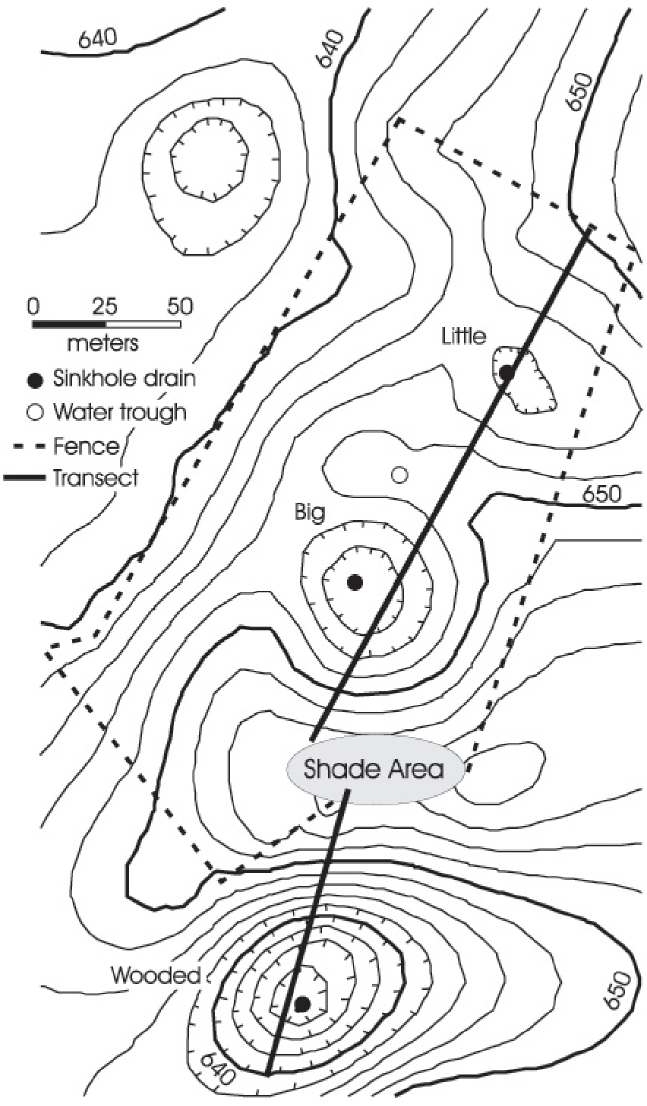

**FIGURE 1.** Contour map of the study site. Contour interval = 2 m.

two sinkholes in permanent pasture on a stocker beef enterprise. The sinkhole pastures are rotationally grazed from March through October each year at a stocking rate of 3.5 head ha$^{-1}$. The pastured (grazed) sinkholes will be referred to as Little and Big sinkholes. The main forage species are tall fescue (*Festuca arundinacea Schreb.*) and white clover (*Trifolium repens L.*). Soil test results show pH above 5.5, so lime is not spread most years. However, the site did receive 4.94 Mg ha$^{-1}$ of lime in the fall of 1994 and in the fall of 2000. Mineral fertilizers N (as ammonium nitrate), P, and K are applied each spring at rates of 34, 100, and 56 kg ha$^{-1}$, respectively. A wooded sinkhole (referred to as the Wooded sinkhole) in the same area and in line with Little and Big sinkholes was also studied. Wooded sinkhole has been excluded from grazing since 1990. Prior to 1990, Wooded sinkhole was unfenced and cattle had free access to it. Vegetation in the Wooded sinkhole consists of trees, shrubs, and forbs. The dominant species in the Wooded sinkhole are shagbark hickory (*Carya ovata* Mill. K. Koch), multiflora rose (*Rosa multiflora* Thunb.), and poison ivy (*Rhus radicans* L.) for trees, shrubs, and forbs groups, respectively. Sinkholes are closed topographic depressions having no external drainage. Karst sinkholes may have visible openings into the underlying conduit system at their lowest elevation, but the sinkholes in this study did not. Little, Big, and Wooded sinkholes lie along a lineament. Sinkhole diameters ranged from 105 to 120 m but vary in depth; therefore, the degree of slope is different. Average slopes as measured from the sinkhole rims to sinkhole floors were 13, 17, and 33% along transects in Little, Big, and Wooded sinkholes, respectively.

Soil samples were collected in late autumn from the three sinkholes at distances that would represent the topography of individual sinkholes. The samples were collected more than 6 months following fertilization with ammonium nitrate. By sampling about 3 weeks after the last cattle rotation and following several killing frosts, we hoped to minimize N dynamics driven by growing plants. Sampling was limited to the root zone or top 20-cm soil depth in order to avoid missed samples at shallow locations and to avoid sampling in the epikarstic zone. The epikarstic zone is the soil and weathered bedrock near the soil-bedrock interface, which has an extremely variable hydrology because of the fracturing, secondary porosity, and surficial topographic characteristics of the bedrock. Sample locations were more widely spaced at the sinkhole rims (rim to rim, transect distances were: 0, 6, 15, 24, 33, 39, 45, 51, 54, 57, 60, 63, 69, 75, 84, 93, and 102 m). At each location along this transect, 9 soil cores (5-cm diameter) were collected within 30 cm of each transect point. Soil cores were subdivided into four depths (0 to 2.5 cm, 2.5 to 5.0 cm, 5 to 10 cm, and 10 to 20 cm) and bulked according to depth to minimize the heterogeneity of the sample. The soil core samples were air-dried and sieved to <2 mm.

Subsamples of soil (0.5 g) were transferred into 50-ml centrifuge tubes along with 30 ml of deionized water. Tubes were shaken for 16 h on a reciprocating shaker at 200 strokes per minute, followed by centrifugation at 10,000 rpm for 15 min. The supernatants were filtered through 0.45-µm filters and frozen until analysis for water-soluble $NO_3$-N and $NH_4$-N. Nitrate and ammonium concentrations in soil water extracts were determined by ion chromatography with suppressed conductivity (Dionex DX 500 I. C., AS14 4-mm anion column and CS14 4-mm cation column).

Cattle grazing behavior was determined by using global positioning systems (GPS) during the 1999 and 2000 grazing seasons. A handheld, survey-grade GPS was used to locate manure patties following each cattle rotation through the pasture in 1999. Each patty received a paint mark following its location determination so the patty would not be located again after subsequent rotations. In 2000, GPS receivers and data loggers mounted on collars were used on two head of cattle to determine cattle locations every 15 min. All GPS data were differentially corrected to submeter accuracy.

## RESULTS AND DISCUSSION

Mean water-extractable $NO_3$-N and $NH_4$-N concentrations and standard deviations (by soil depths) are listed in Table 1. Statistical differences between $NO_3$-N and $NH_4$-N concentrations at each soil depth within each sinkhole were calculated by the paired-*t* option of the TTEST procedure in the Statistical Analy-

**TABLE 1**
**Mean Water Extractable $NO_3$-N and $NH_4$-N Concentrations at Designated Soil Depths**

| | | Soil depth (cm) | | | |
|---|---|---|---|---|---|
| | | 0–2.5 | 2.5–5 | 5–10 | 10–20 |
| Sinkhole | | N (mg kg$^{-1}$ soil) | | | |
| Big | $NO_3$-N | 21.2 (6.3) | 22.8 (13.7) | 16.6 (10.1) | 17.4 (8.1)* |
| | $NH_4$-N | 49.9 (19.1)* | 26.4 (18.4) | 11.1 (14.7) | 8.3 (13.6) |
| Little | $NO_3$-N | 24.1 (3.1) | 21.0 (5.9)* | 20.0 (3.3)* | 23.7 (2.9)* |
| | $NH_4$-N | 37.0 (13.0)* | 8.2 (7.0) | 5.9 (7.9) | 1.3 (2.5) |
| Wooded | $NO_3$-N | 22.6 (11.8) | 15.2 (9.7) | 16.9 (8.0) | 21.5 (5.7) |
| | $NH_4$-N | 43.5 (37.2)* | 35.1 (38.0)* | 22.8 (30.3) | 18.5 (20.6) |

*Note:* Standard deviations appear in parentheses. A statistically higher ($p > 90\%$) concentration of $NO_3$-N or $NH_4$-N at any soil depth within a sinkhole is indicated by an asterisk (*).

sis System. $NH_4$-N concentrations were higher ($p > 90\%$) than $NO_3$-N concentrations in the upper two soil depths in the Wooded sinkhole. $NH_4$-N concentrations were higher ($p > 90\%$) than $NO_3$-N concentrations in the top soil layer and $NO_3$-N concentrations tended to be greater ($p > 90\%$) at the three lower soil depths in the two pasture sinkholes.

The higher proportion of $NO_3$-N and low concentrations of $NH_4$-N at the deeper soil depths in the pasture sinkholes may be a result of N cycling driven by grazing animals and subsequent manure deposition. However, since Wooded sinkhole is not vegetationally similar to the pasture sinkholes a direct comparison on grazing cannot be made. As a cation, $NH_4$-N is more readily adsorbed by soil particles and organic matter and is not easily leached. $NO_3$-N is an anion and is easily leached. Boyer and Pasquarell[10] showed a direct relationship between nitrate concentrations in karst springs of southeastern West Virginia and percent basin in agriculture.

Fig. 2 shows the distribution of water extractable $NO_3$-N and $NH_4$-N, by soil depth, along transects across Big sinkhole (pasture) and the Wooded sinkhole. Results for Big and Little sinkholes were very similar, so Little sinkhole is not discussed in the rest of this paper for simplification. It is noteworthy that N concentrations increased at the center of the pasture (Big) sinkhole. The same trend was not apparent in the Wooded sinkhole. However the Wooded sinkhole had a high concentration of $NH_4$-N in the upper soil layers at its northern edge. The northern end of the Wooded sinkhole transect is within a few meters of a small grove of trees in the pasture sinkhole. Cattle tended to congregate under the trees for shade and the high $NH_4$-N concentration in the Wooded sinkhole may have resulted from transport of fecal material across a bedrock outcropping. The southern end of the transect in Big sinkhole approached the same grove of trees, although more distant, and showed an increase in $NH_4$-N in the upper soil layer. There may be some indication of increased N concentrations near the center of the Wooded sinkhole, but it may only be an artifact of the high variability exhibited across that sinkhole. Redistribution of N by woody vegetation in Wooded sinkhole needs to be considered as an important factor affecting $NO_3$-N and $NH_4$-N distributions across that sinkhole.

Spearman's rank correlation[19] was used to compare $NO_3$-N, $NH_4$-N, and the sum of $NO_3$-N and $NH_4$-N concentrations to presence of grazing livestock at the four soil depths in the pasture sinkholes. A significant correlation ($p > 95\%$) was observed between $NO_3$-N concentrations at the two deeper soil depths and percent of time cattle were within 10 m of the sample sites. The correlation coefficient was 0.48 and 0.38 at the 5- to

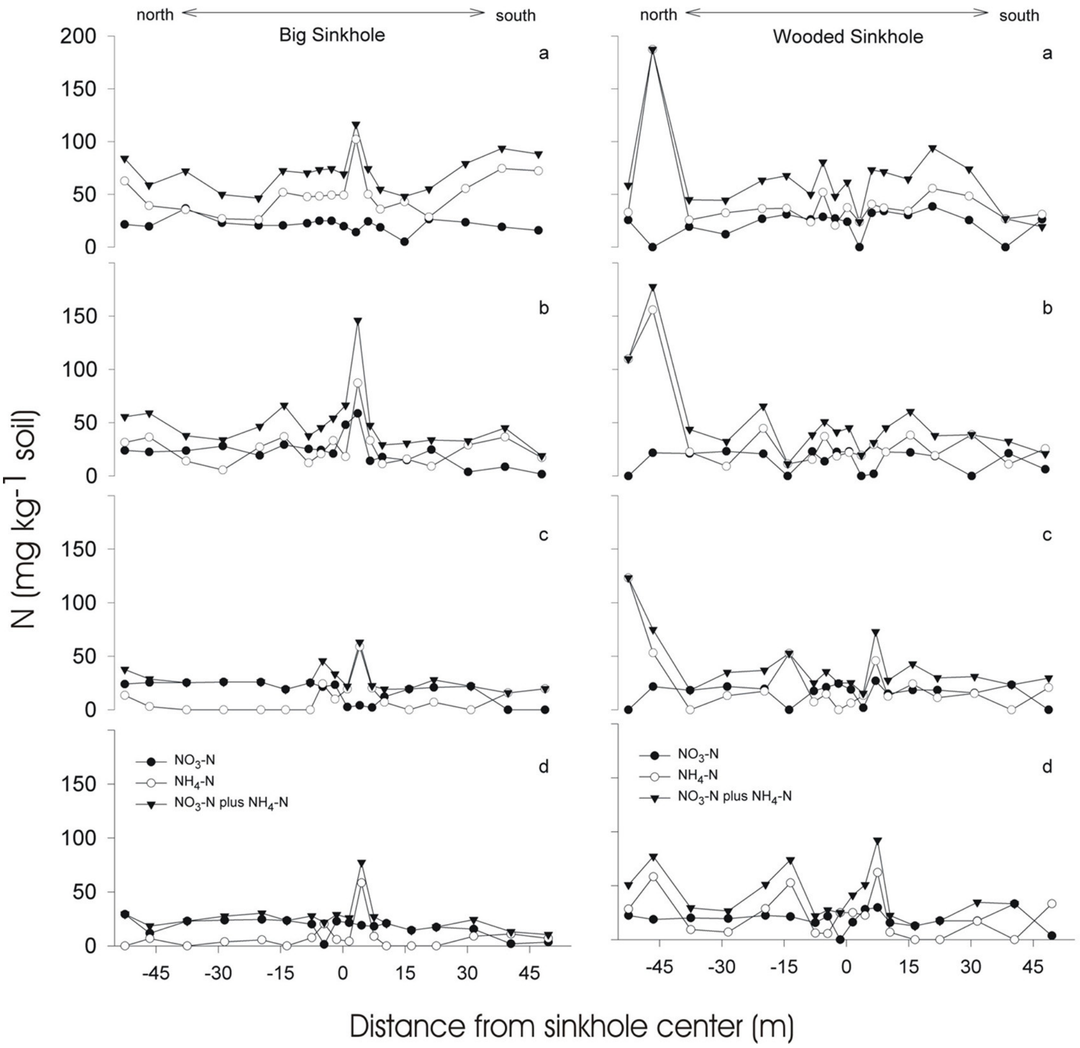

**FIGURE 2.** Mean water extractable $NO_3$-N, $NH_4$-N, and the sum of $NO_3$-N and $NH_4$-N (mg $kg^{-1}$ soil) along transects in Big Sinkhole (pasture) and Wooded Sinkhole (ungrazed since 1989) at soil depths (a) 0 to 2.5 cm, (b) 2.5 to 5 cm, (c) 5 to 10 cm, and (d) 10 to 20 cm.

10-cm soil depth using 1999 and 2000 grazing behavior data, respectively. The correlation was 0.38 at the 10- to 20-cm soil depth for both years. These results indicate that the highest soil $NO_3$-N concentrations at the deeper soil depths were associated with those areas of the pasture receiving the greatest frequency of visitation by the cattle as determined by GPS. Since $NO_3$-N is environmentally important and the deeper soil depth is close to the epikarstic zone, these findings might be important to the development of grazing strategies that protect karst groundwater from leached $NO_3$-N.

There also appears to be a possible redistribution of inorganic N in the sinkhole related to water and soil movement toward the sinkholes' center drains. It is interesting to note that in the pasture (Big) sinkhole, $NH_4$-N tends to be higher than $NO_3$-N in the shallow soil layers, but $NO_3$-N is higher in the deeper layers, except near the sinkhole center (Fig. 2). In that dynamic area of the sinkhole, where cover collapse is frequent, $NH_4$-N is elevated as a result of shallow soil layers, and possibly transported fecal material, mixing with the deeper soil layers.

## CONCLUSIONS

Water-extractable $NO_3$-N and $NH_4$-N were measured along transects at four soil depths in two grazed sinkholes and one wooded sinkhole. Distribution of soil $NO_3$-N and $NH_4$-N might have been related to increased frequency of visitation by cattle and to topographic and hydrologic redistribution of soil and fecal matter in the grazed sinkholes. Karst pastures are characterized by under drainage and funneling of water and contaminants to the shallow aquifer. Control of $NO_3$-N leaching from karst pasture may depend upon management strategies that change livestock grazing behavior in sinkholes and reduce the opportunity for water and contaminants to quickly reach sinkhole drains. Strategies like location of shade, water troughs, and supplemental mineral feeding are important for directing fecal matter away from sinkhole drains. Precision farming is a newer technology that can be used to strategically place lime and fertilizer. Manipulation of botanical composition in grazed sinkholes might be another tool to discourage grazing in sensitive sinkhole areas during hydrologically active seasons.

## ACKNOWLEDGMENTS

We thank D. Carter, E. Cook, D. Hall, and R. Lester for technical assistance and continuous help during the course of the project. The hospitality and cooperation of the Lloyd and Lonnie Burns families was very much appreciated. The use of their pasture and livestock made this project possible.

| | | |
|---|---|---|
| Received: | July | 13, 2001 |
| Revised: | November | 7, 2001 |
| Accepted: | November | 9, 2001 |
| Published: | November | 27, 2001 |
